# Application of recombinant severe fever with thrombocytopenia syndrome virus nucleocapsid protein for the detection of SFTSV-specific human IgG and IgM antibodies by indirect ELISA

**DOI:** 10.1186/s12985-015-0350-0

**Published:** 2015-08-04

**Authors:** Fuxun Yu, Yanhua Du, Xueyong Huang, Hong Ma, Bianli Xu, Ferdinard Adungo, Daisuke Hayasaka, Corazon C. Buerano, Kouichi Morita

**Affiliations:** Department of Virology, Institute of Tropical Medicine, Nagasaki University, 1-12- 4, Sakamoto, Nagasaki 852-8523 Japan; Henan Center for Disease Control and Prevention, Zhengzhou, China

**Keywords:** Severe fever with thrombocytopenia syndrome virus, Recombinant nucleocapsid protein, IgG, IgM

## Abstract

**Background:**

Severe fever with thrombocytopenia syndrome (SFTS) is an emerging disease that was first reported in China in 2011. It is caused by SFTS virus (SFTSV) which is a member of the *Phlebovirus* genus in the *Bunyaviridae* family. SFTSV has been classified as a BSL3 pathogen. There is a need to develop safe and affordable serodiagnostic methods for proper clinical management of infected patients.

**Methods:**

The full length nucleocapsid (N) gene of SFTSV Yamaguchi strain was amplified by RT-PCR and cloned to an expression vector pQE30. The recombinant (r) SFTSV-N protein was expressed by using *Escherichia coli (E. coli)* expression system and purified under native conditions. rSFTSV-N protein based indirect IgG and IgM enzyme linked immunosorbent assay (ELISA) systems were established to detect specific human IgG and IgM antibodies, respectively. One hundred fifteen serum samples from clinically suspected-SFTS patients were used to evaluate the newly established systems and the results were compared with the total antibody detecting sandwich ELISA system.

**Results:**

The native form of recombinant (r) SFTSV-N protein was expressed and purified. Application of the rSFTSV-N protein based indirect IgG ELISA to the 115 serum samples showed results that perfectly matched those of the total antibody sandwich ELISA with a sensitivity and specificity of 100 %. The rSFTSV-N protein based indirect IgM ELISA missed 8 positive samples that were detected by the total antibody sandwich ELISA. The sensitivity and specificity of rSFTSV-N-IgM capture ELISA were 90.59 and 100 %, respectively.

**Conclusions:**

The rSFTSV-N protein is highly immunoreactive and a good target for use as an assay antigen in laboratory diagnosis. Its preparation is simpler in comparison with that used for the total antibody sandwich system. Our rSFTSV-N protein-based IgG and IgM ELISA systems have the advantage of distinguishing two types of antibodies and require small volume of serum sample only. They are safe to use for diagnosis of SFTS virus infection and especially fit in large-scale epidemiological investigations.

## Background

Severe fever with thrombocytopenia syndrome virus (SFTSV), also named as fever, thrombocytopenia and leukopenia syndrome virus (FTLSV) or Huaiyangshan virus, is an emergent virus that was first reported in 2011 [1–3]. The sources of serum samples where the virus was identified were from patients infected in 2009 and 2010 in China. Severe fever with thrombocytopenia syndrome (SFTS), the disease caused by the virus has a major clinical presentations that include fever, thrombocytopenia, leukocytopenia, gastrointestinal symptoms, neurological symptoms, bleeding tendency, as well as less specific clinical manifestations [1, 2]. This disease has a case-fatality rate ranging from 2.5 to 30 % in different areas of endemicity [4]. Human-to-human transmission of SFTSV was reported to occur through close contact with the blood and/or body secretions of infected patients [5–9].

After the first identification of SFTS, SFTS cases have been reported in 13 provinces of China [10]. Recently, the existence of this disease has also been confirmed in Japan and South Korea [11–15]. In Japan, the case-fatality rate of 55 % (6/11) was apparently higher than that in China, where an average of 12 % of cases was fatal [13]. Data on the high fatality rate due to SFTSV indicate that SFTSV is a threat to human health. Another tick-borne phlebovirus, the Heartland virus, which was detected in Missouri, is phylogenetically associated with SFTSV. It causes severe febrile illness with thrombocytopenia, leukopenia in the total blood cell count, and elevated levels of liver enzymes [16].

For the diagnosis of SFTS, laboratory confirmation is essential because the clinical manifestations of SFTS are non-specific. Virus isolation from the blood of viremic patients is the direct evidence of SFTSV infection, however, it is time-consuming and needs high security biocontainment facility [1, 2]. Detection of SFTSV genome could be achieved by different nucleic acid detection techniques such as reverse transcription-PCR (RT-PCR) [1, 2], real-time RT-PCR [14, 17], reverse transcription-loop-mediated isothermal amplification assay (RT-LAMP) [18–20], reverse transcription-cross-priming amplification coupled (RT-CPA) with vertical flow (VF) visualization [21]. Although these techniques have high sensitivity and specificity in early diagnosis, the duration of viraemia in SFTSV infection is very short, generally 1–6 days after the disease onset [22]. Hence, the nucleic acid detecting techniques are applicable only during the acute phase of the disease which is within 1 week after its onset. The final confirmation of infection in many cases may rely on the detection of the specific antibodies to SFTSV.

SFTSV is a member of the *Phlebovirus* genus in the *Bunyaviridae* family. Like other bunyaviruses, the L segment encodes the RNA-dependent RNA polymerase; the M segment has an open reading frame (ORF) coding for a GnGc precursor in the order Gn-Gc; whereas the S segment uses ambisense coding to express two proteins: one is a nucleocapsid (N) protein encoded by the 5′ half of viral complementary sense S RNA, and the other is a nonstructural (NS) protein encoded by viral sense S RNA [1, 2, 23]. Nucleocapsid (N) protein is one of the most immunodominant viral proteins among members of the *Bunyaviridae* family. Recombinant N protein of Rift Valley Fever (RVF) virus, another member of the *Phlebovirus* genus, was reported to be used in a detection system for the laboratory diagnosis of RFV infection in humans and animals [24–26]. In SFTSV, Jiao *et al.* developed a recombinant N protein based sandwich enzyme linked immunosorbent assay (ELISA) for detecting the total antibodies against this virus in humans and animals [27]. In our present report, recombinant SFTSV-N (rSFTSV-N) protein was expressed by using *Escherichia coli (E. coli)* expression system and then purified. rSFTSV-N protein based IgG ELISA and IgM ELISA systems were established. Serum samples from clinically-suspected SFTS patients were used to evaluate the newly established systems and results were compared with those obtained by using the total antibody detecting sandwich ELISA system.

## Results

### Expression and purification of rSFTSV-N protein

**Fig. 1:**
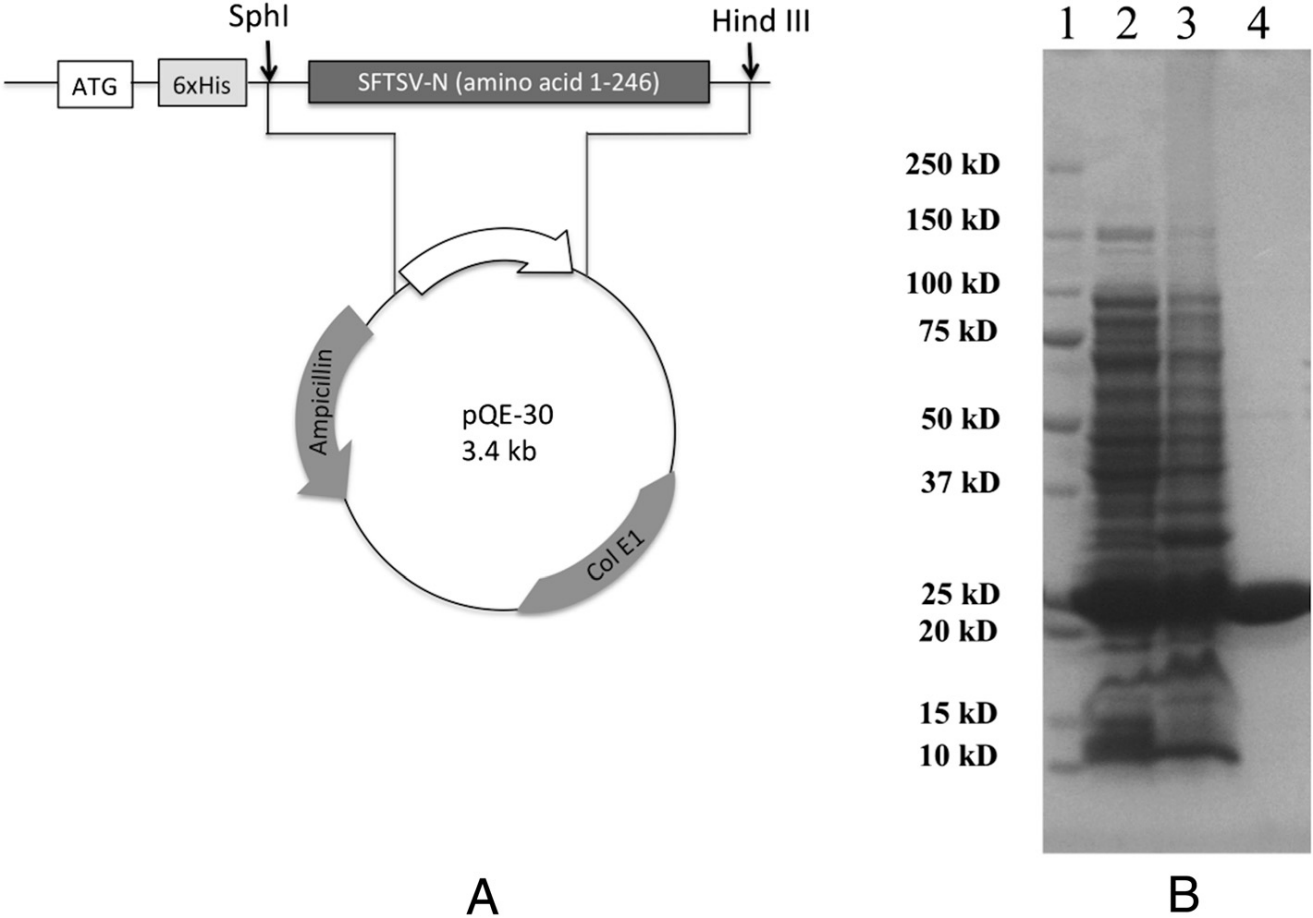
Expression and purification of recombinant SFTSV-N protein. **a** Schematic figure of the recombinant expression plasmid with the SFTSV-N insert. **b** SDS-PAGE analysis of recombinant SFTSV-N protein. The proteins from the supernatant and pellet of the recombinant expressing *E. coli* cell lysate and the purified recombinant protein were analyzed in a 5–20 % gradient SDS-PAGE gel which was stained with Coomassie brilliant blue. Lane 1: protein marker (Precision plus protein standards, Bio-Rad); Lane 2: supernatant of sonicated *E. coli* cell lysate after centrifugation; Lane 3: pellet of sonicated *E.coli* cell lysate; Lane 4: purified recombinant protein

**Fig. 2:**
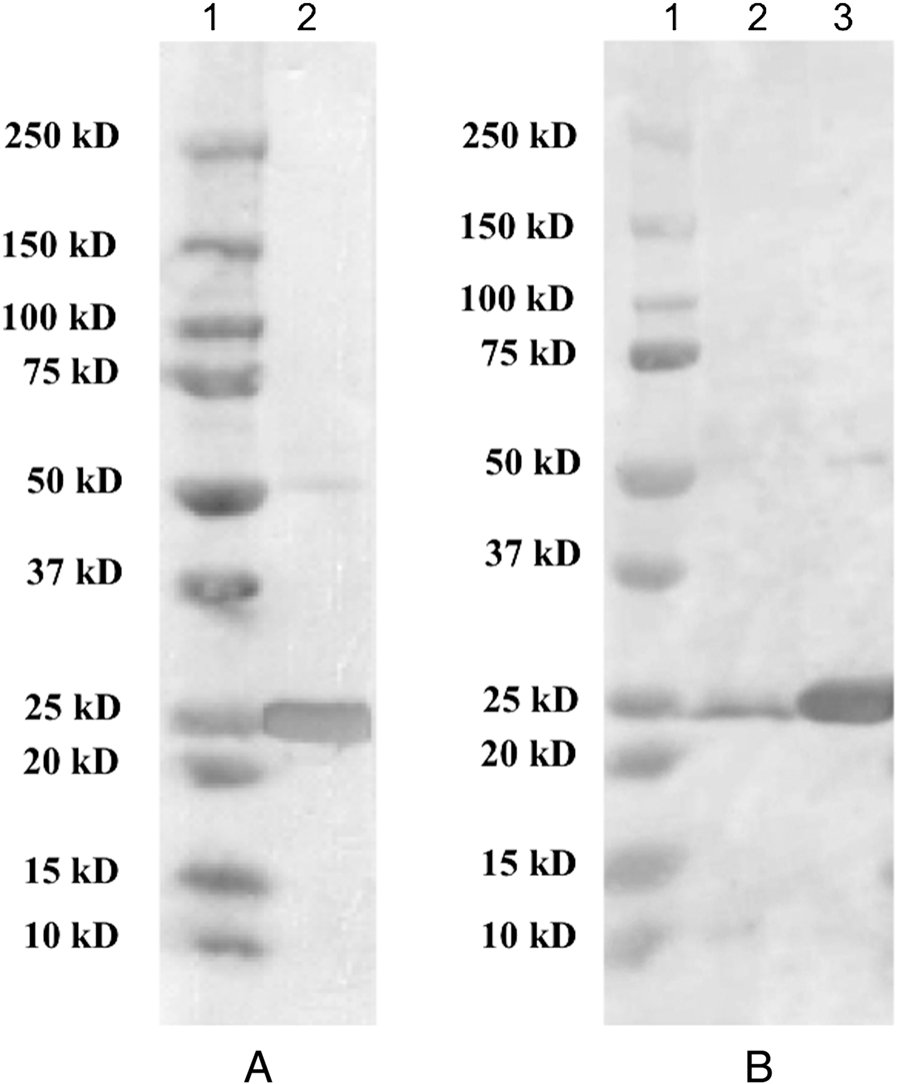
Western-blot analysis of purified SFTSV-N protein. **a** Reactivity of recombinant protein to mouse anti-Histidine antibody. Lane 1: protein marker (Precision plus protein standards); lane 2: purified SFTSV-N protein. **b** Reactivity to SFTS patient serum. Lane 1: protein marker; lane 2: SFTSV infected Vero-E6 cell lysates; lane 3: purified SFTSV-N protein

The SFTSV nucleocapsid gene encoding amino acid residues 1–246 of the full length nucleocapsid protein was successfully amplified by RT-PCR and cloned into an expression vector in frame and downstream of the six-histidine tag (Fig. 1a). The sequence and reading frame of the N gene were confirmed by DNA sequencing of the recombinant plasmid. The recombinant protein was successfully expressed in *E. coli*. Most of the expressed protein was soluble (Fig.1b lane 2). The rSFTSV-N protein was purified from the supernatant under native conditions. Analysis of purified recombinant protein by SDS-PAGE and Coomassie blue staining revealed a single protein band of 26kD as predicted by the amino acid sequence of the nucleocapsid protein (Fig. 1b lane 4). The identity of the rSFTSV-N protein was further confirmed by Western blot assay with mouse monoclonal antibody against histidine and the use of SFTS patient serum sample (Fig. 2).

### Determination of serum dilutions for indirect IgG and IgM

To determine the appropriate dilutions of serum samples for the indirect IgG and IgM ELISAs using the rSFTSV-N protein mentioned above, samples from healthy volunteers and SFTS confirmed patients were diluted two-fold from 1:100 up to 1:1000. After the application of IgG ELISA to 94 serum samples from healthy volunteers, 9 gave an optical density (OD) value of 0.5 at 1:400 and lower dilutions of the samples. At higher dilutions, 1:800 and 1:1000, the OD values of all the samples were between 0.1-0.2. The OD values of two serum samples for SFTS-confirmed patients did not change much; the value was 3.0 at 1:100 dilution and was reduced only to 2.4 at 1:1000 dilution. Based on these results, the dilution of serum samples for IgG ELISA was set at 1:1000 for convenience.

In IgM ELISA, 12 of the healthy volunteer samples gave an OD value of 0.5 in 1:100 and 1:200 dilutions but all samples gave reduced values between 0.1–0.2 at 1:400 dilution. The OD value of two samples from SFTS-confirmed patients was reduced from 2.2 at 1:400 dilution to 0.8 at 1:800 dilution. Thus, the dilution for IgM ELISA was set at 1:400.

At the set serum dilutions, 1: 1000 for IgG and 1:400 for IgM, all the 94 healthy volunteer serum samples gave an OD values between 0.1 and 0.2, hence, the cut-off for giving a negative result was set at 0.4.

### Evaluation of rSFTSV-N protein based indirect IgG ELISA

Among the 115 serum samples from SFTS-suspected patients, 85 samples were positive by rSFTSV-N protein-based indirect IgG ELISA and 30 were negative. The OD value for negative samples ranged from 0.1 to 0.2, and from 0.4–2.6 for positive samples. These results perfectly matched the results obtained by using the total antibody ELISA kit. The sensitivity and specificity of rSFTSV-N-IgG ELISA in comparison with the kit were 100 % (Table 1).

**Table 1:** Sensitivity and specificity of SFTSV-N-IgG ELISA with reference to the total antibody sandwitch ELISA kit

Sandwich ELISA	SFTSV-N-IgG ELISA		Total
	Positive	Negative	
Positive	85	0	85
Negative	0	30	30
Total	85	30	115
Concordance^a^ : 100 % Sensitivity^b^ : 100 % Specificity^c^: 100 %			

^a^(No. of samples positive by both methods + No. of samples negative by both methods)/total number of samples × 100

^b^True positive/(true positive + false negative) × 100

^C^True negative/(true negative + false positive) × 100

### Evaluation of rSFTSV-N protein based indirect IgM ELISA

Among the 115 serum samples from SFTS-suspected patients, 77 samples were positive and 38 were negative by rSFTSV-N protein-based indirect IgM ELISA. The OD values for negative samples ranged from 0.1 to 0.2, and from 0.4–2.2 for positive samples. Our indirect IgM ELISA failed to detect the eight samples that were positive by the total antibody sandwich ELISA kit. Compared with the kit, the sensitivity and specificity of the rSFTSV-N-IgM capture ELISA were 90.59 and 100 %, respectively (Table 2).

**Table 2:** Sensitivity, and specificity of SFTSV-N-IgM ELISA with reference to the total antibody sandwitch ELISA kit

Sandwich ELISA	SFTSV-N-IgM ELISA		Total
	Positive	Negative	
Positive	77	8	85
Negative	0	30	30
total	77	38	115
Concordance^a^ :93.04 % Sensitivity^b^ :90.59 % Specificity^c^:100 %			

^a^(No. of samples positive by both methods + No. of samples negative by both methods)/total number of samples × 100

^b^True positive/(true positive + false negative) × 100

^C^True negative/(true negative + false positive) × 100

## Discussion

In the present study, we expressed rSFTSV nucleocapsid protein in *E. coli* and purified the recombinant protein to near homogeneity by the his-tag based affinity chromatography under native conditions and used it as an assay antigen in the indirect IgG and IgM ELISA.

Nucleocapsid protein is the most abundant protein in many viruses and recombinant nucleocapsid protein has been used for the sero-diagnosis of many viruses like, SARS and Nipah viruses [28, 29]. In the *Phlebovirus* family, RVF virus N protein is a good target for IgG and IgM ELISA systems [24–26]. For SFTS virus, there has been only one report on the development of an ELISA system (double-antigen sandwich assay system) which makes use of a recombinant N protein for detecting total antibodies and this system was validated by neutralization test [27]. The total antibody ELISA kit used in the present study was based on this system. In contrast to the preparation of the recombinant N protein used in this antigen sandwich assay system, our recombinant protein was mostly soluble and was purified under native condition without using any detergent thereby skipping the arduous work of refolding the denatured protein. The expression and purification procedures described in this study provide a simple and efficient way to obtain pure SFTSV N protein in large quantity.

Using this rSFTSV-N protein, we developed indirect IgG and IgM ELISA for human serum, and compared with a commercial total antibody detection sandwitch ELISA kit. Evaluation of 115 serum samples from SFTSV-suspected patients showed a concordance rate of 100 and 93.04 % for the IgG and IgM ELISA in comparison with the total antibody detection sandwitch ELISA kit, respectively. The sensitivity and specificity of the rSFTSV-N protein based indirect IgG ELISA system were both 100 % with regard to total antibody detection sandwitch ELISA kit (Table 1). The sensitivity and specificity of the rSFTSV-N protein based indirect IgM ELISA system were 90.59 % and 100 % respectively, with regard to total antibody detection sandwitch ELISA kit (Table 2). The indirect IgM ELISA system failed to catch eight samples that were positive by the total antibody kit. This may be caused by the lower sensitivity of indirect IgM ELISA method or the IgM antibody against SFTSV did not exist in these samples. All the 115 serum samples were collected from SFTS suspected patients who recovered after their illness and all the positive samples have the specific IgG (Table 1). It is well known that the IgG competes with the IgM in binding to antigen thereby reducing the sensitivity of indirect IgM ELISA [30]. Still our indirect IgM ELISA has a sensitivity of 90.59 %; it is an acceptable level for clinical diagnosis, especially for use in developing countries. For more sensitive diagnosis method, we are currently developing an IgM capture ELISA system.

The total antibody sandwich-ELISA system which was developed by Jiao *et al.* was widely used in China because it is simple to perform, could be used to human and all kinds of animals. But the disadvantages of this method are that it cannot distinguish IgG from IgM and that more volume of the serum (serum used without dilution) is required for the assay thus, limiting its application for clinical diagnosis and large scale epidemiological studies. Our SFTSV-N protein based systems detect IgG and IgM separately, so it can distinguish previous or recent infection, respectively. The serum dilution is 1:1000 for IgG and 1:400 for IgM; this greatly save the serum used and will be quite beneficial for precious samples and large scale epidemiological studies.

The rSFTSV-N protein based indirect IgG and IgM ELISA systems presented here eliminate the use of infectious virus in the antigen production, which requires high level of microbiological security facilities. Hence they are safer methods for diagnosis. The expression and purification procedure for recombinant SFTSV-N protein is simple and easy allowing an easy standardization of the antigen production. The advantages of using a prokaryotic host to produce recombinant SFTSV-N protein would be considerable due to the ease of scale-up, and the low costs involved in growing bacteria. It would be especially useful in cases of large-scale epidemiological investigation and for application in developing countries.

## Conclusion

In conclusion, the rSFTSV-N protein is highly immunoreactive in human infection and it is a good target for laboratory diagnosis. Our rSFTSV-N protein-based IgG and IgM ELISA systems are safe, specific and sensitive tools for serological diagnosis of SFTS virus infections and especially fit to for use in large-scale epidemiological investigations.

## Materials and methods

### Serum samples

Two serum samples from SFTS-confirmed patients collected in 2014 and 94 serum samples from healthy volunteers collected in 2004–several years before the earliest identified SFTS patient was reported–were used as positive and negative controls, respectively, in determining the serum dilution for the IgG and IgM indirect ELISA we developed in the present study. To evaluate these two assays, 115 serum samples collected in 2013 in Henan Province, China were used in this study. These samples were collected from patients who recovered from an illness that was suspected to be SFTS.

### Virus inoculation and RNA extraction

The Yamaguchi strain of SFTSV (GenBank accession no. AB817995, AB817987, and AB817979) that was isolated in 2013 from a patient in Yamaguchi Prefecture, Japan was inoculated to confluent monolayer of Vero-E6 cells. These cells were then maintained at 37 °C in Eagle’s Minimum Essential Medium supplemented with 2 % fetal calf serum and 0.2 mM of each non-essential amino acids for 5 days. The infected culture fluid (ICF) was harvested and from a 140 μl of this ICF, viral RNA was extracted using the QIAamp viral RNA mini kit (Qiagen, Hilden, Germany) according to the manufacturer’s instructions. The extracted RNA was eluted in 60 μl of elution buffer and then used as template for RT-PCR.

### Construction of recombinant plasmid

RT-PCR was performed by using the primers 5’- GGAGCATGCATGTCGGAGTGGTCCAGG-3’ and 5’-AATAAGCTTTTACAGGTTTCTGTAAGCA-3’ to generate the full length N gene of SFTSV. Sense and reverse primers contained SphI and Hind III restriction sites (underlined), respectively. The PCR amplified DNA fragments were digested with SphI and Hind III, purified by a QIAEX II gel extraction kit (Qiagen, Hilden, Germany), and subsequently cloned into the corresponding restriction site of the pQE30 vector (Qiagen, Hilden, Germany). The insert of recombinant plasmid was confirmed to be in frame by DNA sequencing. The expression construct encompassing amino acid (aa) 1–246, the full length of SFTSV N protein with a vector derived His-tag (histidine hexmer) at the N-terminus, was obtained. The resultant recombinant protein was designated as rSFTSV-N protein.

### Expression and purification of the recombinant SFTSV- N protein

The rSFTSV-N protein was expressed by inserting the recombinant plasmid containing the SFTSV-N sequence into *E. coli* strain *XL-1 blue* and cultured at 37 °C in Luria-Bertani (LB) medium containing 100 μg/ml of ampicillin. When the optical density (OD 600 nm) of the culture reached 1.0, the expression of recombinant protein was induced for 3 h by the addition of 0.2 mM isopropyl β-D-thiogalactoside (IPTG). After harvest by centrifugation, the *E. coli* pellet was washed in phosphate buffered saline solution (PBS), then resuspended in 10 mM PBS pH 7.5 with 500 mM NaCl and frozen at −80 °C. After freezing and thawing three times, the cell suspension was sonicated for 2 min with an interval of 1 s between pulses and centrifuged at 30,000 g for 15 min at 4 °C. The supernatant was then applied to a Talon™ IMAC resin column (Clontech, USA). After being washed with a binding buffer (10 mM PBS with 500 mM NaCl containing 20 mM imidazole, pH 7.5), the purified protein was eluted with an elution buffer (10 mM PBS with 500 mM NaCl containing 250 mM imidazole, pH 7.5). The protein solution was aliquoted and stored in a final concentration of 10 % glycerol at −80 °C until use. Protein concentrations were determined by the Bradford method using a Bio-Rad protein assay reagent kit (Bio-Rad, USA), and the purity of the protein was analyzed by sodium dodecyl sulfate-polyacrylamide gel electrophoresis (SDS-PAGE).

### Western blot analysis

Western blot analysis was performed as described before [28]. Briefly, protein marker (Precision plus protein standards, Bio-Rad), SFTSV infected Vero-E6 cell lysates and the purified recombinant protein were separated in a 5–20 % gradient polyacrylamide gel (ATTO Corporation, Japan) before being electrotransferred onto a PVDF membrane (Immobilon, Millipore, USA) by using a semidry electroblotter (Sartorius, Germany). The membrane was blocked with Blockace (Yokijirushi, Sapporo, Japan) overnight at 4 °C to prevent nonspecific staining, then subjected to reaction with mouse anti-histidine (GE Healthcare Life Sciences,1:1000 dilution), or patient serum sample (1:100 dilution) for 1 h at 37 °C before incubation with rabbit anti-mouse IgG, or goat anti-human peroxidase conjugate (American Qualex, Califonia, USA,1:1000 dilution) for 1 h at 37 °C. Finally, the reaction was visualized by dimethyl aminobenzidine (DAB) staining.

### Determination of serum dilution for indirect IgG and IgM

Serum samples from two SFTS-confirmed patients and from 94 healthy volunteers were diluted at 1:100, 1:200, 1:400, 1:800 and 1:1000 dilutions. The diluted sera were checked by rSFTSV-N based indirect IgG and IgM ELISA separately as described below. The appropriate dilution of the serum samples for these two assays was determined based on OD values at 410 nm.

### ELISA procedures using the recombinant nucleocapsid protein

To evaluate the usefulness for diagnosis of the rSFTSV-N protein we developed, we established an indirect IgG and IgM ELISA for the laboratory diagnosis of SFTSV infection in humans with the serum samples as clinical specimens. These assays have common procedures which will be described here and the procedures specific for each assay will be described under each assay. The plates used were 96-well Nunc immunoplates (Thermo Scientific, Denmark), and all the reagents were in 100 μL volumes. The optimal concentration of rSFTSV-N protein used to coat the microplates was determined by checkerboard titration with reference serum samples (2 from SFTS-confirmed patients and 2 from healthy volunteers). The coating buffer was 0.01 M PBS, pH 7.4, and plate coating was conducted at 4 °C overnight. Wash buffer was 0.01 M PBS with 0.1 % (vol/vol) Tween 20 (PBS-T). Plates were washed three times with PBS-T after exposure to a specific reagent at each step of the procedure except at the last step as described below. Dilution of all serum samples and reagents were in 5 % nonfat milk (Difco, Detroit, USA) in PBS-T. Incubations, except for substrate, were done for 1 h at 37 °C. Plates with 100 μL H_2_O_2_-ABTS substrate (Kirkegaatrd & Perry, Gaithersburg, MD) in each well were incubated for 30 min at 37 °C. The plates were read spectrophotometrically and OD values at 410 nm were recorded.

#### Indirect IgG ELISA

In the indirect IgG ELISA, 96-well plates were subjected to the following steps: coating of each well with 50 ng rSFTSV-N protein per well followed by human serum samples that were diluted 1:1,000 in 5 % nonfat milk in PBS-T, then detection of bound IgG with 1:30,000 diluted horseradish-peroxidase-conjugated goat anti-human IgG (American Qualex, Califonia, USA) which was made visible after adding H_2_O_2_-ABTS substrate, the last reagent in this series of procedure. OD values at 410 nm were recorded on a microplate spectrophotometer. Each serum sample was tested in duplicate, and the mean OD for each sample was calculated. Reference serum samples were run in every assay. The mean OD of a sample more than twice the mean OD of the negative control serum was considered positive.

#### Indirect IgM ELISA

The procedure for indirect IgM ELISA was similar to IgG ELISA. The changes were that the serum samples were diluted at 1:400 and the detection of bound IgM was donewith 1:10,000 diluted horseradish peroxidase-conjugated goat anti-human IgM (American Qualex, Califonia, USA).

### Detection of total SFTSV antibody

Serum samples from suspected SFTS patients were subjected to a commercial ELISA kit (Xinlianxin Biomedical Technology CO., LTD, Wuxi, Jiangsu, China) following the manufacturer’s protocol. The kit is a double-antigen sandwich enzyme-linked immunosorbent assay kit that detects total antibodies including IgG and IgM against SFTSV [27]. Results obtained by using this total antibody ELISA kit was compared to the results obtained by applying the IgG and IgM indirect ELISA described above.

### Ethical consideration

This research was approved by the Institutional Review Board at the Center for Disease Control and Prevention of Henan Province. All participants gave written informed consent for the use of their serum samples for research purposes.

## Abbreviations

SFTSV: Severe fever with thrombocytopenia syndrome virus
RT-PCR: Reverse transcription-polymerase chain reaction
rSFTSV-N: Recombinant SFTSV nucleocapsid
*E. coli*: *Escherichia coli*
ELISA: Enzyme linked immunosorbent assay
ICF: Infected culture fluid
PBS: Phosphate buffered saline solution
PBS-T: PBS-Tween-20
SDS-PAGE: Sodium dodecylsulfate polyacrylamide gel electrophoresis
